# PLGA nanoparticles loaded with recombinant antimicrobial protein PIP significantly improves the survival state and pathological damage caused by ETEC O8-induced sepsis in mice

**DOI:** 10.3389/fvets.2026.1728161

**Published:** 2026-03-13

**Authors:** Xian Li, Jianjie Li, Pengfei Qiu, Ying Zhang, Chunjiang Wang, Menglong Yue, Congshang Lei, Miao Yin, Xuefeng Qi, Xiwen Chen

**Affiliations:** 1 Animal Disease Prevention and Control, Healthy Breeding Engineering Technology Research Center, Mianyang Normal University, Mianyang, Sichuan, China; 2 College of Veterinary Medicine, Northwest A&F University, Yangling, Shaanxi, China; 3 Key Laboratory of Ruminant Disease Prevention and Control (West), Ministry of Agriculture and Rural Affairs, Yangling, China; 4 Hebei Veyong Pharmaceutical Co., Ltd., Shijiazhuang, Hebei, China

**Keywords:** antibiotic alternatives, ETEC O8, PLGA-PIP nanoparticles, sepsis, treatment effect

## Abstract

**Introduction:**

Peptide-based antimicrobial drugs are promising alternatives to antibiotics owing to their broad-spectrum bactericidal activity and unique pathogen membrane disruption mechanism. Our previous study demonstrated that the recombinant antimicrobial protein PIL22-PBD-2 (PIP) inhibits pathogens and repairs intestinal cell damage *in vitro*, but its *in vivo* therapeutic potential against bacterial infections remains uncharacterized.

**Methods:**

In this study, we developed an oral drug delivery nano-platform composed of PIP and poly(lactic-co-glycolic acid) (PLGA) using the double emulsion solvent evaporation method, and evaluated its therapeutic efficacy in a mouse model of sepsis induced by enterotoxigenic *Escherichia coli* O8 (ETEC O8).

**Results:**

PLGA-PIP nanoparticles were successfully prepared and showed excellent resistance to trypsin degradation as well as good biocompatibility *in vivo*. In septic mice, treatment with 300 mg/kg PLGA-PIP significantly alleviated weight loss and clinical symptoms (*p* < 0.05), reduced serum biochemical indices and organ indexes (*p* < 0.05), and decreased ETEC O8 loads in feces, liver, spleen, and kidneys (*p* < 0.01). PLGA-PIP also mitigated pathological damage in major organs, increased duodenal villus height and VH/CD ratio (*p* < 0.05), upregulated the expression of tight junction proteins (ZO-1, E-cadherin) and endogenous antimicrobial factors (Cryptdin-1, Reg3γ) (*p* < 0.01), and suppressed the expression of pro-inflammatory cytokines IL-6, IL-1β, and TNF-α (*p* < 0.01).

**Discussion:**

These findings demonstrate that PLGA-PIP effectively ameliorates ETEC O8-induced sepsis in mice by enhancing intestinal barrier function, reducing pathogen burden, and inhibiting inflammation. Therefore, PLGA-PIP represents a promising oral antibiotic alternative for the treatment of bacterial infections.

## 1 Introduction

Intestinal pathogenic *Escherichia coli* (*E. coli*) is one of the most common causes of bacterial diarrhea in livestock farming, leading to neonatal diarrhea and post-weaning diarrhea (PWD) in piglets (1, 2). After pathogenic *E. coli* infection of the body, bacteria-induced sepsis involves a series of reactions, including the increased secretion of pro-inflammatory mediators, expression decreased of adhesion molecules, and multiple organ dysfunction (3). Moreover, *E. coli* endotoxins often cause disruptions of tight junction integrity, increased intestinal permeability, intestinal malnutrition, and intestinal epithelial cell apoptosis, which leading to impaired intestinal barrier function (2, 4). Damage to the intestinal barrier is not conducive to the absorption of nutrients, which can result in weight loss and facilitate the escape of pathogens from the intestines into the circulation. Additionally, the release of toxins by pathogenic *E. coli* can cause uncontrolled inflammatory reactions and immune dysfunction (5). The underlying mechanism is that the toxins, mainly lipopolysaccharide, are recognized by toll-like receptors, which then activate downstream cell signaling pathways such as nuclear factor-kappa B (NF-κB) and mitogen-activated protein kinase (MAPK). Activated NF-κB and MAPK pathways, in turn, lead to further inflammatory responses, resulting in the production of more pro-inflammatory cytokines (such as IL-6, IL-1β, TNF-α) and immune suppression (6, 7). In veterinary clinical practice, antibiotics are still an important means for the prevention and control of pathogenic *E. coli*. However, the overuse and inappropriate use of antibiotics in livestock farming have led to the emergence of serious antibiotic resistance and residues (1). Therefore, new prevention and control strategies against multi-drug resistant *E. coli* are urgently needed in the livestock farming industry.

Antimicrobial peptides (AMPs) are promising antibiotic alternatives due to their broad-spectrum antibacterial activity (including against drug-resistant bacteria) and unique antibacterial mechanism. Most AMPs act as positively charged amphiphilic molecules that electrostatically adsorb to bacterial membranes, penetrate, and disrupt membrane structures, making it difficult for bacteria to develop resistance (8, 9). Based on this, our team designed the recombinant antimicrobial protein PIL22-PBD-2 (PIP), which integrates functional domains derived from interleukin 22 (IL-22, a cytokine that regulates intestinal epithelial repair and antimicrobial peptide production) (10–12) and porcine β-defensin 2 (PBD2). Our previous *in vitro* study confirmed that PIP has multiple functions: it exhibits direct antibacterial activity against multi-drug resistant ETEC O8, exerts anti-inflammatory effects, and effectively protects IPEC-J2 cells from ETEC O8-induced damage (13). However, as an antimicrobial protein, PIP faces inherent challenges for *in vivo* application: poor oral bioavailability, instability under physiological conditions, susceptibility to interference from anionic substances, and easy degradation by intestinal proteases (9, 14). These limitations severely restrict its therapeutic efficacy and translation from *in vitro* activity to *in vivo* application.

Nanomedicine, particularly nanoparticle technology, can encapsulate various payloads such as RNA, proteins, and drugs, leading to significant advancements in vaccine preparation and tumor treatment (15–17). It has paved the way for the development of peptide-based drugs as nano preparations. Poly (lactic-co-glycolic) acid (PLGA) is a type of biodegradable polymer material, synthesized by copolymerizing lactic acid and glycolic acid monomers (18). It has excellent biocompatibility, degradability and controlled drug release rate, making it widely used in drug delivery, medical devices, and tissue engineering (19). PLGA nanoparticles can be prepared using various techniques, with precipitation and emulsification being the most commonly used methods (18). For encapsulating hydrophilic molecules, peptides, proteins, and nucleic acids, the emulsification method is more suitable (20). Additionally, nanoparticles prepared using the emulsification method may lead to the production of either nanospheres or nano-capsules architectures of nanoparticles (18). An oral drug delivery system composed of PLGA nanoparticles and peptide drugs provides several advantages over free peptides, such as improved bioavailability, reduced toxicity, resistance to gastrointestinal digestion, and enhanced therapeutic effects (14, 21). Therefore, it is reasonable to design a safe peptide-based antimicrobial drug delivery system utilizing the potential delivery properties of PLGA.

Although our previous *in vitro* study confirmed PIP’s potential (13), its *in vivo* therapeutic efficacy against ETEC O8 infection remains unclear, and whether PLGA encapsulation can enhance its delivery and therapeutic effects *in vivo* requires further investigation. Thus, this study utilized a double emulsification method to construct an oral PLGA-PIP nano-delivery system and evaluated its therapeutic effects in a mouse model of ETEC O8-induced sepsis.

## 2 Materials and methods

### 2.1 Animals and ethics declarations

The animal protocol was approved by the Experimental Animal Management and Ethics Review Executive Committee, Northwest A&F University (No. XN2023-0602). Specific pathogen-free (SPF) 6-week-old Kunming mice were obtained from the experimental animal center at Northwest A&F University.

### 2.2 Protein, bacterial strain and cell line

The His-tagged PIP was expressed in our laboratory using the *Pichia pastoris* (*P. pastoris*) strain GS115 (Invitrogen, United States). The *P. pastoris* GS115 and expression vectors pPIC9k (Invitrogen, United States) were routinely available in our laboratory. ETEC O8 (resistant to kanamycin, ampicillin, streptomycin and ofloxacin) isolated from the feces of clinically diarrheal piglets and maintained in our laboratory (13). The strain was cultured at 37 °C with agitation at 150 rpm in nutrient broth. Intestinal porcine epithelial cell line-J2 (IPEC-J2) was obtained from the China Center for Type Culture Collection in Wuhan, and is maintained in liquid nitrogen with a mixture of 90% dimethyl sulfoxide (DMSO) and 10% fetal bovine serum (FBS, PAN, Germany). IPEC-J2 was cultured in Dulbecco’s modified Eagle’s medium (DMEM, Hyclone, United States) containing 10% FBS (PAN, Germany) and 100 IU/mL penicillin as well as 100 μg/mL streptomycin (Gibco, CA, United States) at 37 °C in a 5% CO_2_ atmosphere.

### 2.3 Preparation and characterization of PLGA-PIP

PIP-loaded PLGA nanoparticles (PLGA-PIP) were prepared using the double emulsification volatilization method as described previously (22), with slight modifications. Briefly, 1% (w/v) PLGA (40 mg) was dissolved in a dichloromethane solution as the oil phase, and 1 mL of phosphate-buffered saline (PBS) or 8 mg of PIP dissolved in 1 mL of PBS was used as the first water phase. Then, in an ice bath, sonication was conducted using a sonicator (Scientz Biotechnology, Ningbo, China) in continuous mode for 5 min (pulses of 5 s with 3-s gaps) at an output power of 300 W. The primary emulsion was further emulsified with a secondary water phase (10 mL of 2.0% w/v PVA). Using the same settings as in the previous step, ultrasound was performed for 8 min in an ice bath. Subsequently, the compound emulsion was placed in a 40 °C water bath, and the volatile organic solvent was stirred by magnetic force for 4 h. The precipitate was collected by centrifugation at 12,000 rpm for 30 min at 4 °C, washed 2–3 times with deionized water.

The average diameter and relevant parameters of the PLGA and PLGA-PIP were measured using dynamic light scattering (DLS) with a Malvern Nano-ZS 90 laser particle size analyzer (Malvern Instruments, Royston, United Kingdom). Next, the nanospheres were diluted with sterile water to a concentration of 10 mg/mL and 20 μg/mL, placed on a silicon wafer, allowed to dry naturally, and then sprayed with gold for scanning electron microscopy (SEM) observation (JSM-IT700HR, JEOL, Japan).

The encapsulation efficiency (EE) and loading efficiency (LE) of PLGA-PIP were determined using the previously described method (23). After centrifugation, the amount of PIP encapsulated in PLGA nanoparticles was determined by measuring the protein remaining in the supernatant using a bicinchoninic acid (BCA) protein assay (Beyotime, China). Briefly, a 25 mg/mL bovine serum albumin (BSA) standard stock solution was prepared by dissolving 30 mg BSA in 1.2 mL protein standard diluent, and then diluted to 0.5 mg/mL as the working standard. BCA working solution was prepared by mixing BCA Reagent A and Reagent B at a volume ratio of 50:1. For the assay, 0–20 μL of the 0.5 mg/mL BSA standard (supplemented to 20 μL with diluent, corresponding to final concentrations of 0–0.5 mg/mL) or 20 μL of test sample (supplemented with diluent if necessary) was added to a 96-well plate. After adding 200 μL of BCA working solution to each well, the plate was incubated at 37 °C for 20–30 min. Absorbance at 562 nm was measured using a microplate reader, and the protein concentration of samples was calculated based on the BSA standard curve and the actual sample volume added.

The EE and LE were calculated according to formulas (1) and (2): (1) EE = (A − B)/A × 100%; (2) LE = (A − B)/C × 100%. Where A, B, and C refer to the weight of total PLGA-PIP used and PLGA-PIP nonencapsulated and PIP-loaded PLGA, respectively (*n* = 3).

The release of PIP from the nanoparticles was measured as described previously (24), with slight modifications. Briefly, 13 mL of artificial gastric fluid (pH 1.2, United States Pharmacopeia, USP) containing 130 mg of PLGA-PIL22-PBD2 was divided into 2 mL EP tubes in equal volumes (1 mL each), including two stages as follows: the release was accomplished in 1 mL simulated artificial gastric fluid (pH 1.2, USP) for 2 h in the first stage, and PLGA-PIL22-PBD2 was transferred into intestinal fluid (pH 6.8, USP) for 4 h in the second stage. While stirring at 37 °C, one tube was randomly taken for centrifugation at 0 h, 0.5 h, 1 h, 1.5 h, and 2 h, respectively, to determine the release of PIP in the supernatant. Subsequently, centrifuge the remaining eight tubes and resuspend PLGA-PIP with 1 mL of artificial intestinal fluid. Centrifuge one tube at 0 h, 0.5 h, 1 h, 1.5 h, 2 h, 2.5 h, 3 h, 3.5 h, and 4 h, and measure the release of supernatant protein. The protein content in the supernatant was measured by the BCA assay method within the specified time. The release efficiency (RE) was calculated according to formula (3): RE = D/B × 100%, where D and B refer to the weight of PIP in the supernatant and carried, respectively (*n* = 3).

### 2.4 Western blot and indirect immunofluorescence analysis

To further determine whether PIP was loaded onto PLGA, the protein was detected using western blotting and indirect immunofluorescence. The supernatant of the centrifuged compound emulsion and the resuspended PLGA-PIP were added to 5× protein loading buffer and boiled for 10 min for SDS-PAGE and western blot analysis. Gels were stained with Coomassie brilliant blue R-250. Additionally, the gels were transferred onto a PVDF membrane (Millipore, Burlington, MA, United States). After transfer, the membrane was blocked with 5% skim milk diluted in Tris-buffered solution containing Tween-20 (TBST) for 2 h. After washing with TBST, the blocked membrane was incubated with the mouse anti-His-tag monoclonal antibody (at a dilution of 1:5,000; Abbkine Scientific Co., Ltd., Wuhan, China) for 2 h. Subsequently, the membrane was rewashed with PBST and incubated overnight with a 1:2,000 dilution of horse radish peroxidase (HRP)-conjugated goat anti-mouse IgG antibody (Abbkine Scientific Co., Ltd., Wuhan, China) at a 1:5,000 dilution. The PVDF membrane was washed again with TBST, subsequently stained with ECL Super Sensitive Kit (Dining Biotechnology Co., Ltd., Beijing, China), and finally imaged by the Mini Chemiluminescence imager 610 (Sage Creation Science Co., Ltd., Beijing, China).

The IPEC-J2 monolayer was inoculated with PIP, PLGA, and PLGA-PIP at a concentration of 0.1 mg/mL each for 1 h. The cells were washed 5 times with PBS and then fixed with 4% paraformaldehyde. After fixation, IPEC-J2 cells were blocked with 5% bovine serum albumin diluted in phosphate-buffered solution (PBS) for 2 h. Subsequently, IPEC-J2 cells were incubated with the mouse anti-His-tag monoclonal antibody (1:5,000 dilution; Abbkine Scientific Co., Ltd., Wuhan, China) diluted in a 5% bovine serum albumin solution overnight. They were then incubated with FITC-conjugated goat anti-mouse IgG (Yeasen, China; 1:200). Fluorescence was visualized using confocal laser microscopy (Nikon, Japan).

### 2.5 Stability determination of PLGA-PIP after trypsin treatment

To investigate whether PLGA could protect the activity of PIP from trypsin-induced damage, Oxford cup antibacterial experiments were conducted to determine changes in the bacteriostatic activity of PIP and PLGA-PIP before and after treatment with bovine pancreatic trypsin (specific activity: 300 U/mg; Sigma, United States) *in vitro*, using ETEC O8 as the indicator bacterium. Briefly, 90 mm culture dishes with 20 mL Luria-Bertani (LB) agar medium per dish were used. The bacterial strains were incubated at 37 °C for 12 h in LB medium. Subsequently, 200 μL of suspension containing 10^6^ CFU/mL of bacteria was spread on LB agar medium. An Oxford cup was placed on the surface of the LB agar medium and gently pressed to ensure tight contact with the medium. Finally, 100 μL of 0.5 mg/mL PIP, 3 mg/mL PLGA and 3 mg/mL PLGA-PIP (equivalent to 0.45 mg/kg PIP) either treated with trypsin followed by inactivation with a specific bovine trypsin inhibitor (Sigma, United States) or without trypsin treatment was added into the Oxford cup. The petri dishes were incubated at 37 °C for 10 h, and the antagonistic activity was estimated by the size of the bacteriostatic ring. The experiment was repeated three times under the same conditions to provide error estimates.

### 2.6 Safety evaluation of PLGA-PIP in mice

A total of 20 6-week-old SPF female Kunming mice (23 g–25 g) were randomized into four groups (control group, 75 mg/kg PLGA-PIP group, 150 mg/kg PLGA-PIP group, and 300 mg/kg PLGA-PIP group equivalent to 45 mg/kg PIP; *n* = 5). Four groups of Kunming mice were fed continuously for 14 days with doses of 75 mg/kg, 150 mg/kg, and 300 mg/kg of PLGA-PIP, as well as normal saline. The daily weight changes of Kunming mice were recorded. After 14 days of administering PLGA-PIP, blood was collected from the ophthalmic venous plexus of the mice and analyzed using the BIOBASE BK-400 automatic biochemical analyzer to measure serum creatinine (CRE), aspartate aminotransferase (AST), and urea (UREA) levels. Subsequently, the heart, liver, spleen, lung, and kidney were collected from dissected mice and fixed with 4% paraformaldehyde to prepare tissue sections for HE staining.

### 2.7 Development of mouse sepsis model

A total of 20 SPF-grade female Kunming mice, aged 6 weeks and with comparable body weights (23 g–25 g), were randomly divided into four groups. On the 4th day, except for the control group, the other three groups of mice were intraperitoneally injected with 0.2 mL bacterial solution at different concentrations: 5 × 10^8^ CFU/mL, 1 × 10^9^ CFU/mL, and 2 × 10^9^ CFU/mL, respectively. The control group received an intraperitoneal injection of an equal volume of sterile saline. The mice were observed continuously for 7 days, and the clinical symptoms of the mice, including their fur state, body weight changes, mental state, appetite, feces, and death, were recorded daily. Each group of mice was scored for clinical symptoms to evaluate the severity of inflammation according to a previous method (25). Briefly, no clinical signs, 0; slight clinical signs, 1; moderate clinical signs, 2; and severe clinical signs, 3.

### 2.8 Experimental design and sample collection

**Figure 1:**
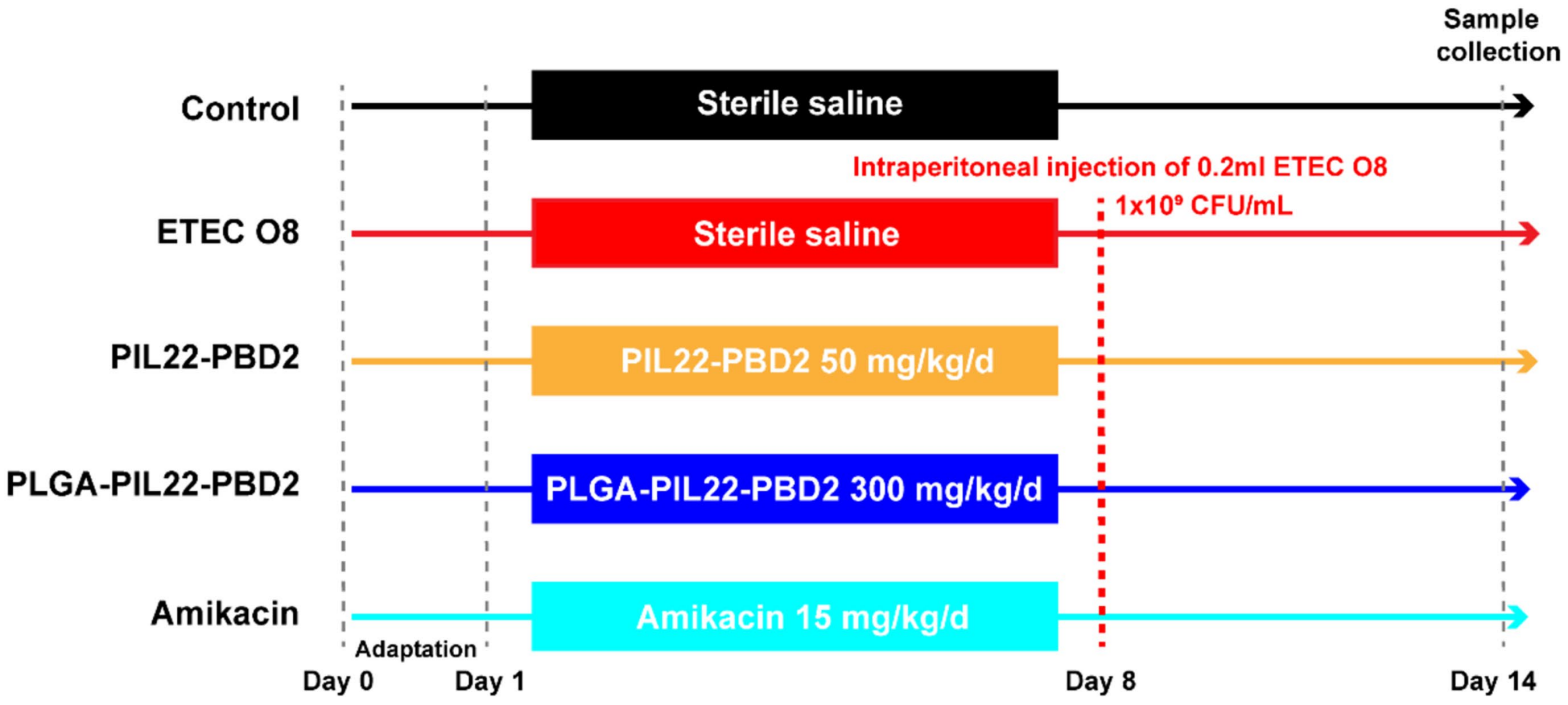
Schematic illustration of the experimental design of the PLGA-PIP treatment in mice infected with *ETEC* O8. All mice had access to water and feed *ad libitum*. PIP and PLGA-PIP were fed daily at a predetermined dose throughout the 14-day trial. In addition to the control group, each mouse in the remaining four groups received an intraperitoneal injection of *ETEC* O8 bacterial solution on the morning of the 8th day. Amikacin treatment was intraperitoneally injected at the recommended dose of 15 mg/kg after bacterial attack in the Amikacin group, while no treatment was given in the *ETEC* group. PIP, PIL22-PBD-2; *ETEC* O8, enterotoxigenic *Escherichia coli*; CFU, colony forming units.

Twenty-five female Kunming mice (23 g–25 g) were randomly assigned to one of the following five groups: (1) the control group (healthy mice were given sterile saline alone); (2) the ETEC O8 challenge group (untreated); (3) the 50 mg/kg PIP group; (4) the 300 mg/kg PLGA-PIP group; (5) the Amikacin group (15 mg/kg). Groups (3) and (4) were fed PIP daily at a predetermined dose throughout the 14-day trial. Notably, the doses of free PIL22-PBD2 (PIP) and PLGA-encapsulated PIL22-PBD2 (PLGA-PIP) administered in this study were strictly designed based on equal amounts of active PIP protein, with dosage adjustment performed according to the measured loading efficiency (LE, 15.08% ± 0.35%) of the PLGA-PIP nano-formulation. In addition to the control group, each mouse in the remaining four groups received an intraperitoneal injection of ETEC O8 bacterial solution on the morning of the 8th day. Amikacin treatment was intraperitoneally administered at the recommended dose of 15 mg/kg after bacterial infection in the Amikacin group, while no treatment was given in the ETEC group. A schematic diagram of the experimental design is shown in Figure 1. Weight changes in each group of mice were recorded daily. The clinical symptoms of the mice were recorded and scored every day after being challenged with ETEC O8. On the 7th day after being challenged with ETEC O8, mouse sera were used for CRE, AST, UREA, glutamine transpeptidase (GGT), and lactate dehydrogenase (LDH) analysis. After euthanasia for cervical dislocation, sterile collection of rectal contents (approximately 500 mg) and organ samples for ETEC O8 load counting. Their hearts and lungs, livers, spleens, and kidneys were weighed and their organ indices calculated. The organs were then fixed with 4% paraformaldehyde for hematoxylin-eosin (H&E) staining analysis. The segments of the duodenum samples were cut separately. One segment was rinsed with PBS and then fixed in a 4% (w/vol) paraformaldehyde solution for further H&E staining analysis. The other segment of the duodenum was placed into sterile tubes, rapidly snap-frozen in liquid nitrogen, and kept at −80 °C until mRNA levels were determined. Rectal content (approximately 500 mg) and organ samples were collected for ETEC O8 load counting.

### 2.9 Rectal content and organ samples bacterial count

The mice were euthanized, aseptically collecting their rectal contents, liver, spleen, and kidney. Use the dilution counting method on a clean bench to determine and calculate the bacterial count of the rectal contents and organs (25). Vortex a mixture of 0.5 g of feces in 4.5 mL of sterile physiological saline and spread the appropriately diluted rectal content on LB agar plates containing penicillin (100 μg/mL) and streptomycin (100 μg/mL) for screening ETEC O8. In addition, add varying volumes of PBS based on the weight of the tissue, and plate the appropriately diluted 100 μL of tissue onto LB agar plates containing penicillin (100 μg/mL) and streptomycin (100 μg/mL) for colony counting. Finally, the plates were incubated in a 37 °C incubator for 12 h, and the results are shown as CFU/g feces and tissues.

### 2.10 Hematoxylin-eosin staining

Toxicological assessment was conducted on the heart, liver, spleen, lungs, and kidneys of each group of mice fed with PLGA-PIP. Pathological evaluation was performed on the liver, spleen, kidneys, and duodenum of each group of mice after intraperitoneal injection of ETEC O8. The small tissue segments mentioned above were fixed in 4% paraformaldehyde and embedded in paraffin. 5-μm thickness sections were obtained from the paraffin blocks by Leica RM2235 microtome (Leica, Germany) and adhered to the slides. After deparaffinization in xylene, sections were stained with hematoxylin-eosin (H&E; Sigma, United States) followed by image capture. Images were captured using a Ni-U microscope with a DS-U3 Image-Pro system (Nikon, Minato, Japan). Villus height and crypt depth were determined using an image processing and analysis system (Image-Pro Plus 6.0, United States), and a previously described calculation method was adopted (3). Pathological damage to the liver was scored according to morphological criteria, with the severity of ballooning degeneration as the primary evaluation index (graded 0 to 3 points: 0 points = no damage, 1 point = mild damage, 3 points = severe damage). Pathological damage to the spleen was scored based on the degree of inflammatory cell infiltration (0 points = no inflammatory cell infiltration, 1 point = mild inflammatory cell infiltration, 3 points = severe inflammatory cell infiltration). Pathological damage to the kidney was scored as follows: 0 points = normal morphological structure with no damage, 1 point = mild edematous damage, 3 points = severe edematous damage.

### 2.11 RNA extraction and qPCR

Total RNA samples from the duodenum were purified and reverse transcribed into cDNA using the Cellular RNA AutoExtraction Kit-DeepWell Plate (Magnetic Beads) (Kangma-Healthcode, Shanghai, China) and PrimeScript™ RT reagent Kit with gDNA Eraser (Takara Biotechnology Co., Ltd., Kusatsu, Japan). Then, the cDNA in 20 μL Trans Start^®^ Top Green qPCR Super Mix (Trans Gen Biotech, China) was amplified by qRT-PCR using the Light Cycler 96 real-time PCR system (Bio-rad, United States) with the designed primers listed in Table 1. The reaction procedure included a pre-denaturation stage (95 °C, 5 min), a 40 cycles PCR stage (denaturation in 95 °C for 15 s, anneal in 60 °C for 30 s, extension in 72 °C for 30 s). The melting curve is obtained by gradually warming from 65 °C to 95 °C. Levels of mRNA were calculated using $2^{-\Delta \Delta C_{T}}$ method and normalized to those of β-actin mRNA.

**Table 1:** Primers for $2^{-\Delta \Delta C_{T}}$ method qRT-PCR in this study.

Gene name	Primer sequences	Origin of sequences
IL-6	F: CCCCAATTTCCAATGCTCTCC	(42)
	R: CGCACTAGGTTTGCCGAGTA	
TNF-α	F: AGCACAGAAAGCATGATCCG	(42)
	R: CCACAAGCAGGAATGAGAAGAGG	
IL-1β	F: TTCAGGCAGGCAGTATCACTCATTG	(43)
	R: TGTCGTTGCTTGGTTCTCCTTGTAC	
ZO-1	F: CGCCTTCATACAATAAAGCAAATCATAG	(42)
	R: ACTGCGCATAATTAAGACGATCAAC	
E-cadherin	F: CGCCTTCATACAATAAAGCAAATCATAG	NM_009864
	R: ACTGCGCATAATTAAGACGATCAAC	
Cryptdin-1	F: CTAGTCCTACTCTTTGCCCT	NM_010031.2
	R: TTGCAGCCTCTTGATCTACA	
Reg3γ	F: CCATCTTCACGTAGCAGC	NM_011260.2
	R: CAAGATGTCCTGAGGGC	
β-actin	F: GTGACGTTGACATCCGTAAAGA	(42)
	R: GTAACAGTCCGCCTAGAAGCAC	

### 2.12 Statistical analysis

All data were expressed as means ± SEMs of at least three independent assays. Statistical analyses were carried out using GraphPad Prism 8.0 (GraphPad Software, Inc., San Diego, CA, United States). Differences between groups were analyzed by Student’s *t*-test or one way ANOVA. *p* < 0.05 was considered statistically significant. Lesion scores were analyzed using non-parametric tests. Mann–Whitney *U* test was applied for two-group comparisons, and Kruskal–Wallis *H* test followed by Dunn’s post-hoc test was used for multiple-group comparisons (GraphPad Prism 8.0, La Jolla, CA, United States). *p* < 0.05 was considered statistically significant.

## 3 Results

### 3.1 Preparation of PLGA and PLGA-PIP

**Figure 2:**
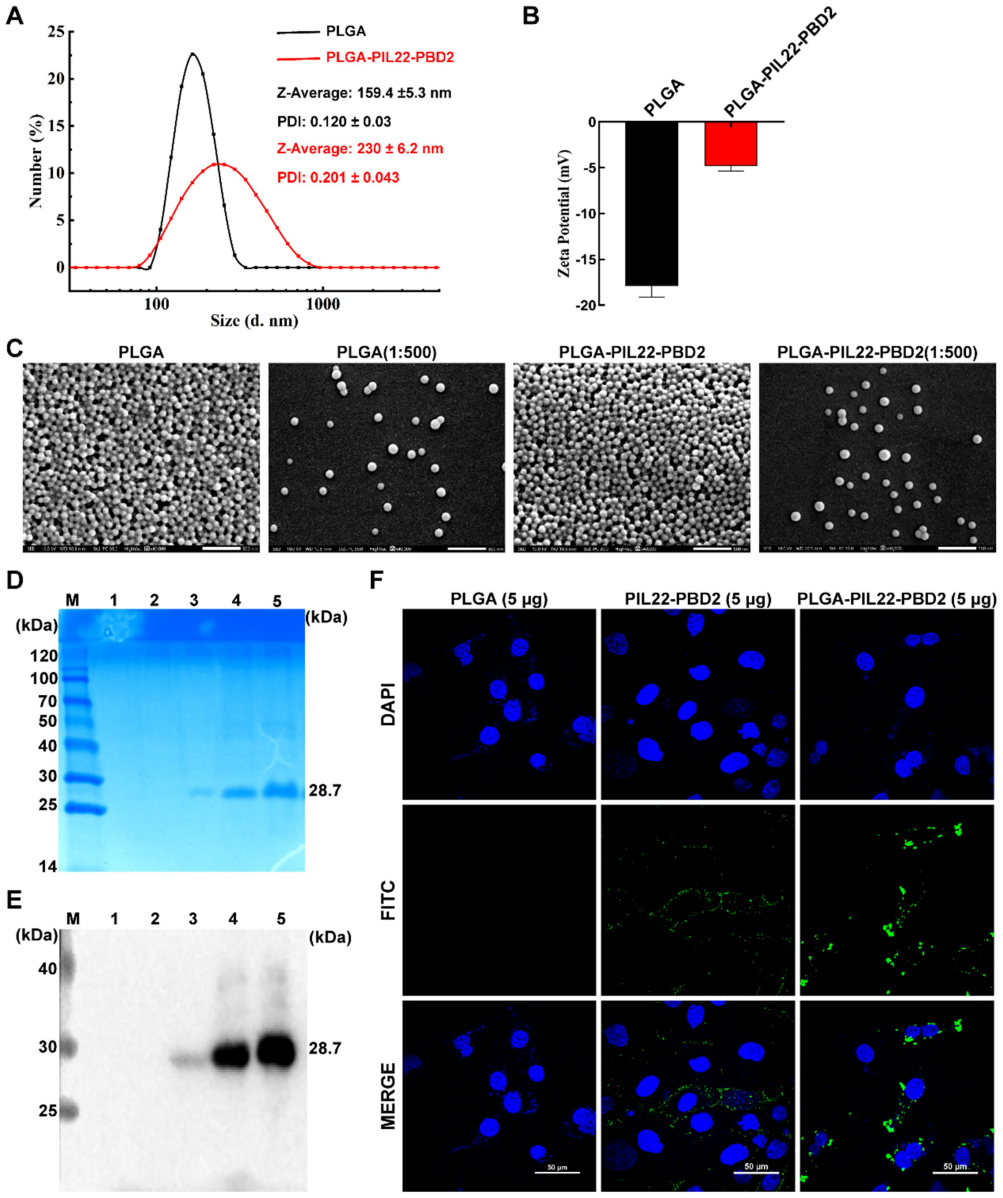
Preparation of PLGA-PIP. **(A)** The particle size distribution of PLGA and PLGA-PIP. **(B)** Zeta potential of PLGA and PLGA-PIP; values are means ± SEMs, *n* = 3. **(C)** Representative scanning electron microscope image of PLGA and PLGA-PIP. **(D)** SDS-PAGE and **(E)** WB analyses of PLGA-PIP. Lane M: protein marker. Lane 1: the supernatant obtained from centrifuging a PLGA solution. Lane 2: the PLGA solution was centrifuged to produce precipitation; Lane 3: the supernatant obtained from centrifuging a PLGA-PIP solution. Lanes 4 and 5: the PLGA-PIP solution was centrifuged to produce precipitation. **(F)** Representative images showing indirect immunofluorescence identification of PLGA-PIP on IPEC-J2 cells. PDI, polydispersity index; PIP, PIL22-PBD-2.

PLGA nanoparticles (PLGA) and PLGA-PIP were prepared using the double emulsification volatilization method. The average particle size distribution and zeta potential of PLGA and PLGA-PIP were evaluated. The results as showed in Figure 2A reveal that the size of PLGA-PIP is larger compared with that of PLGA, indicating that PIP was loaded into the PLGA channels and surface. In addition, PLGA and PLGA-PIP have similar polydispersity index (PDI), the smaller the PDI, the more stable the system (14). Zeta potential of PLGA was −17.9 ± 0.7 mV, and PIP layer was added to the PLGA shell to form PLGA-PIP, then the surface potential turned into −4.8 ± 0.3 mV (Figure 2B). The scanning electron microscope (SEM) analysis show that both the diluted and undiluted PLGA-PIP nanoparticles are in a regular spherical shape (Figure 2C). To further determine whether PIP was fully loaded, SDS-PAGE analysis, western blot, and indirect immunofluorescence were carried out. By conducting SDS-PAGE analysis, bands associated with PIP were found in both the resuspended PLGA-PIP and the supernatant of the centrifuged compound emulsion (Figure 2D). Through western blot and HIS antibody identification, the band was determined to be PIP, as the C-terminus of PIL22-PBD contains a HIS tag (Figure 2E). In addition, indirect immunofluorescence in IPEC-J2 cells also confirmed that naked PIP and PLGA-loaded PIP were recognized by specific antibodies, while PLGA was not (Figure 2F). Therefore, these results imply that PLGA and PLGA-PIP were successfully prepared.

### 3.2 Characterization of PLGA-PIP

**Figure 3:**
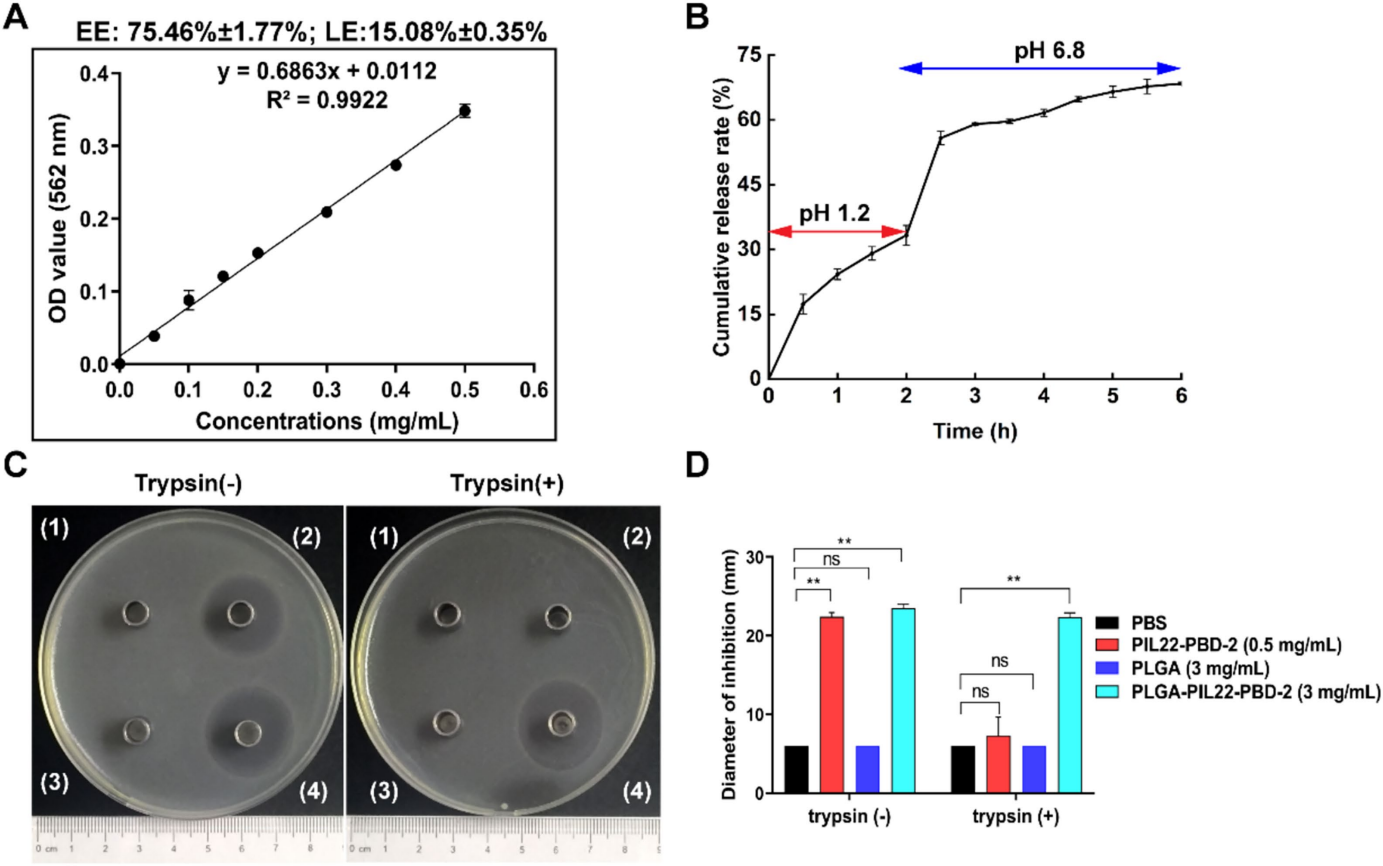
Release of PIP from PLGA-PIP in artificial gastric and intestinal fluids, as well as PLGA-PIP resistance to activity damage induced by trypsin. **(A)** PIP content detection standard curve. The encapsulation efficiency (EE) and loading efficiency (LE) of PLGA for PIP are 75.46% ± 1.77 and 15.08% ± 0.35%, respectively. These values represent means ± SEMs from *n* = 3 samples. **(B)** The continuous release of PIP from PLGA-PIP in solution (pH 1.5 and 6.8) for 6 h, values are means ± SEMs, *n* = 3. **(C)** Representative bacteriostasis plates of PBS (1), PIP (2), PLGA (3), and PLGA-PIP (4) against *E. coli* ETEC O8. Trypsin (+) and trypsin (−) represent that the experimental drugs above are treated with and without trypsin, respectively. **(D)** Statistical analysis of inhibition zone data for the aforementioned experimental drugs with and without trypsin treatment. The differences between the groups were determined by one-way ANOVA followed by Tukey’s *post-hoc* analysis. ^*^p < 0.05 and ^**^p < 0.01. PIP, PIL22-PBD-2.

To investigate the release of PIP from PLGA-PIP over time, we conducted *in vitro* tests simulating the pH environments of artificial gastric juice and intestinal fluid. Firstly, we established standard curve for the detection of PIP. Using the standard curve, we determined that the encapsulation efficiency (EE) and loading efficiency (LE) of PLGA-PIP were 75.46% ± 1.77% and 15.08 ± 0.35%, respectively (Figure 3A). Subsequently, the release of PIP from PLGA was measured in artificial gastric fluid (pH 1.2) and intestinal fluid (pH 6.8), simulating the continuous release process *in vivo* after oral administration (14). The release curve shows that the cumulative release rate of PIP was 68% within6 hours (Figure 3B). With the change in release medium and corresponding pH increase from 1.2 to 6.8 (over 2 h to 2.5 h), the cumulative release rate of PIP reached 33.28 and 35.10% in artificial gastric fluid and intestinal fluid, respectively (Figure 3B).

To further determine the bacteriostatic properties of PLGA-PIP under trypsin treatment, the activity of PIP was assessed using the Oxford Cup bacteriostasis test. As shown in Figures 3C,D, there was no bacteriostatic circle observed when normal saline was used as a negative control (Figure 3C) (1). Both PIP and PLGA-PIP without trypsin treatment exhibited good antibacterial activity and produced a 20 mm bacteriostatic zone (Figure 3C) (2), (4). As expected, PLGA showed no activity against ETEC O8 (Figure 3C) (3). It was gratifying that PLGA-PIP still exhibited antibacterial activity when exposed to trypsin during treatment, which indirectly demonstrates that PLGA can protect PIP from degradation to some extent under these experimental conditions.

### 3.3 Biocompatibility evaluation of orally administered PLGA-PIP *in vivo*

**Figure 4:**
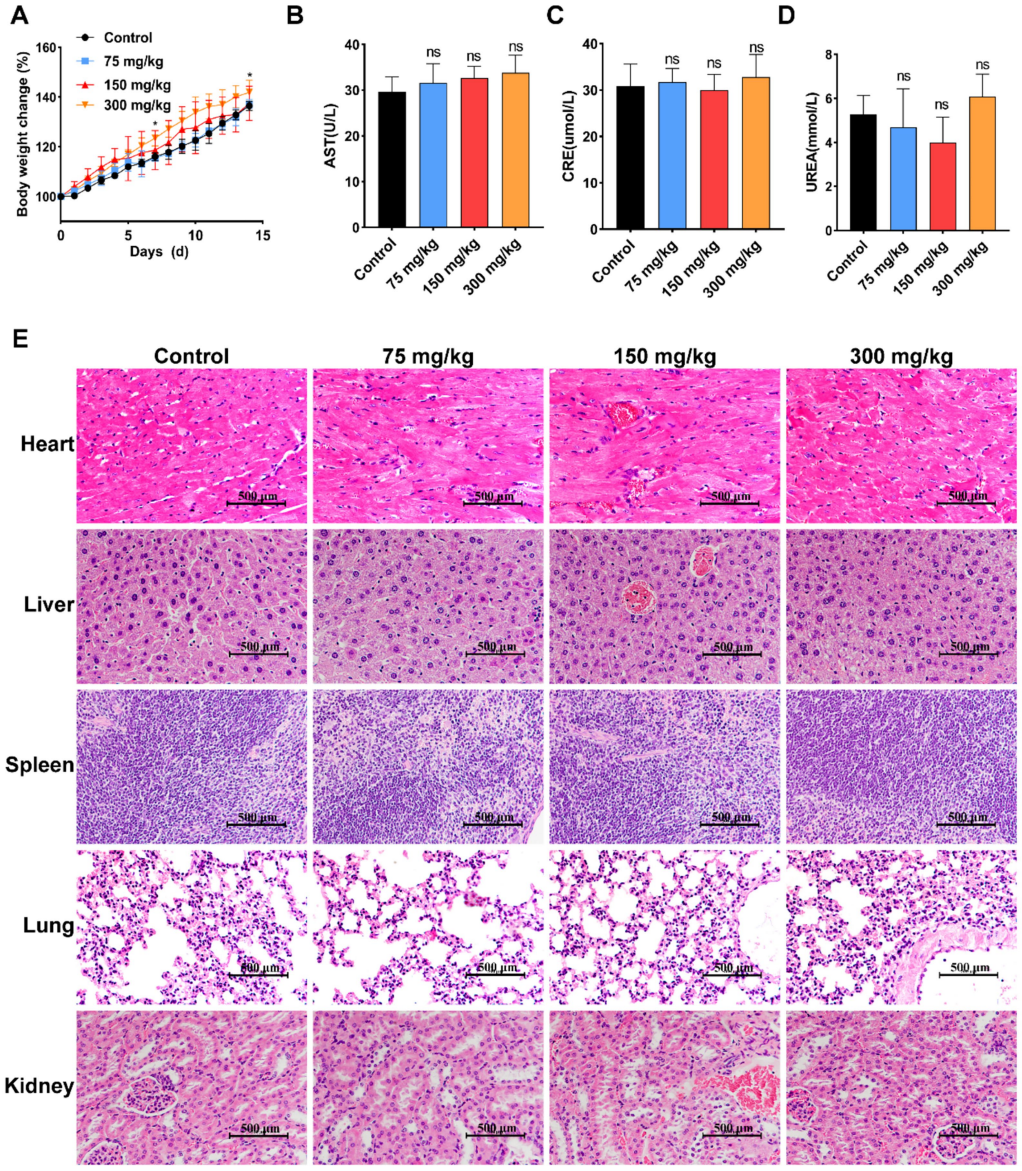
Biocompatibility evaluation of orally administered PLGA-PIP in mice. **(A)** Changes in body weight of mice during the trial period. **(B)** AST, **(C)** CRE, and **(D)** urea levels in serum of mice treated with the PLGA-PIP; values are expressed as means ± SD, *n* = 5. **(E)** Histopathological morphology analysis of the heart, liver, spleen, lungs, and kidneys in mice after administration of different concentrations of PLGA-PIP for 14 days. Scale bar: 500 μm. The differences between the groups were determined by Student’s *t*-test analysis. ns represents no significant difference, ^*^p < 0.05 vs. control group.

Body weight, biochemical indices, and histopathological analysis of organs were used as indicators to initially examine the potential toxicity and safety profiles of different concentrations of PLGA-PIP in Kunming mice after continuous oral administration for 14 consecutive days. As shown in Figure 4A, compared to the control group, mice given 300 mg/kg PLGA-PIP showed a significant increase in body weight (*p* < 0.05). Furthermore, serum AST, UREA, and CRE levels in indicated group were normal compared to the control group (Figures 4B–D). After administration, the heart, liver, spleen, lung, and kidney tissues were excised for histopathological analysis. Comparing with the normal group, no significant typical pathological changes were observed in PLGA-PIP treated group with different concentration (Figure 4E). These results indicate that the administration of 300 mg/kg, the therapeutic dose of PLGA-PIP, not only did not induce significant organ damage but also significantly increased the weight of the mice. Based on the above results, a dose of 300 mg/kg of PLGA-PIP was administered for the treatment of mice infected with ETEC O8 to ensure optimal therapeutic efficacy.

### 3.4 Establishment of the sepsis model in mice

**Figure 5:**
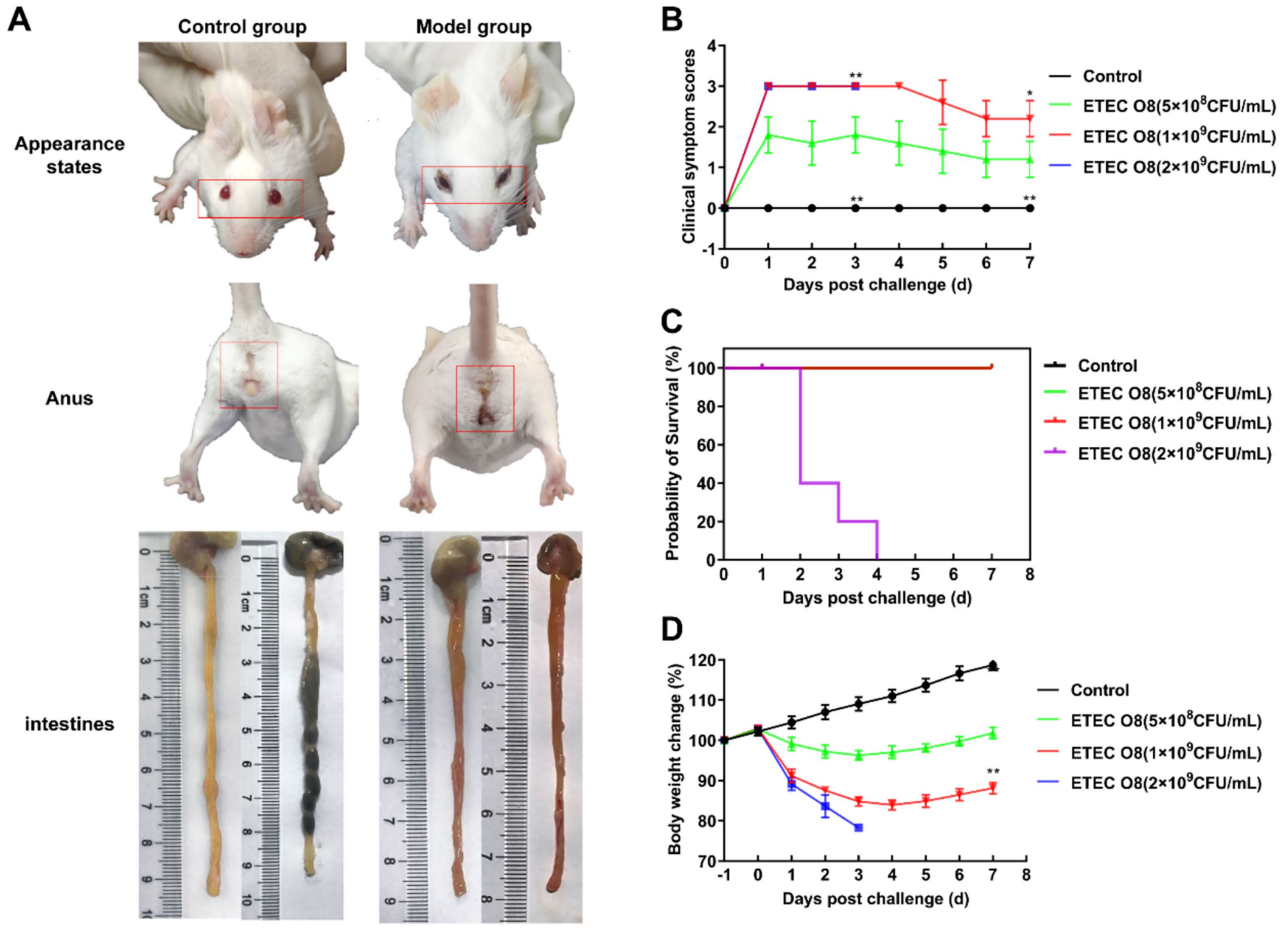
Establishment of the sepsis model in mice. **(A)** The appearance changes of the perianal area and the intestines of the control group and the model group mice were compared. **(B)** Clinical symptom scoring of mice infected with different doses of *E. coli* O8. **(C)** Intraperitoneal injection of different dosages of *E. coli* O8 in mice influences their survival rates. **(D)** Changes in body weight of mice infected with different doses of *E. coli* O8. Error bars represent mean ± SD, *n* = 5. The differences between the groups were determined by one-way ANOVA followed by Tukey’s *post-hoc* analysis. ^*^p < 0.05 and ^**^p < 0.01 vs. E O8 (5 × 10^8^ CFU/mL) group.

Three concentrations of ETEC O8 were used to induce infection in mice, and clinical symptoms, weight changes, and mortality were monitored to determine the optimal amounts of bacteria needed to establish an inflammation model. On day 1, mice injected intraperitoneally with ETEC O8 showed symptoms of mental depression, messy hair, swollen conjunctiva with secretions, and mice in the medium and high dose groups developed diarrhea (Figure 5A). Clinical symptoms of mice in each group were continuously observed for 7 days, and the results showed that the mice infected with ETEC O8 at 5 × 10^8^ (CFU/mL) showed higher score compared to other treated group (Figure 5B). Additionally, all mice in the high-dose bacterial challenge group died 2 days after infection, whereas the mice in the other groups did not die during the entire experimental period (Figure 5C). In terms of weight change, the trend of weight loss in the low-dose bacterial challenge group mice began to reverse on the third day, and the mice showed improved mental state, increased appetite, and obvious disappearance of conjunctival swelling and secretions. Conversely, the severity of clinical symptoms and weight loss trend in mice infected with medium dose ETEC O8 were higher than those in mice infected with low dose ETEC O8 (Figures 5B,D). Based on these findings, a medium dose of ETEC O8 is more suitable for establishing a mouse model of inflammatory disease.

### 3.5 PLGA-PIP significantly improves survival status and alleviates bacterial infection in sepsis mice

**Figure 6:**
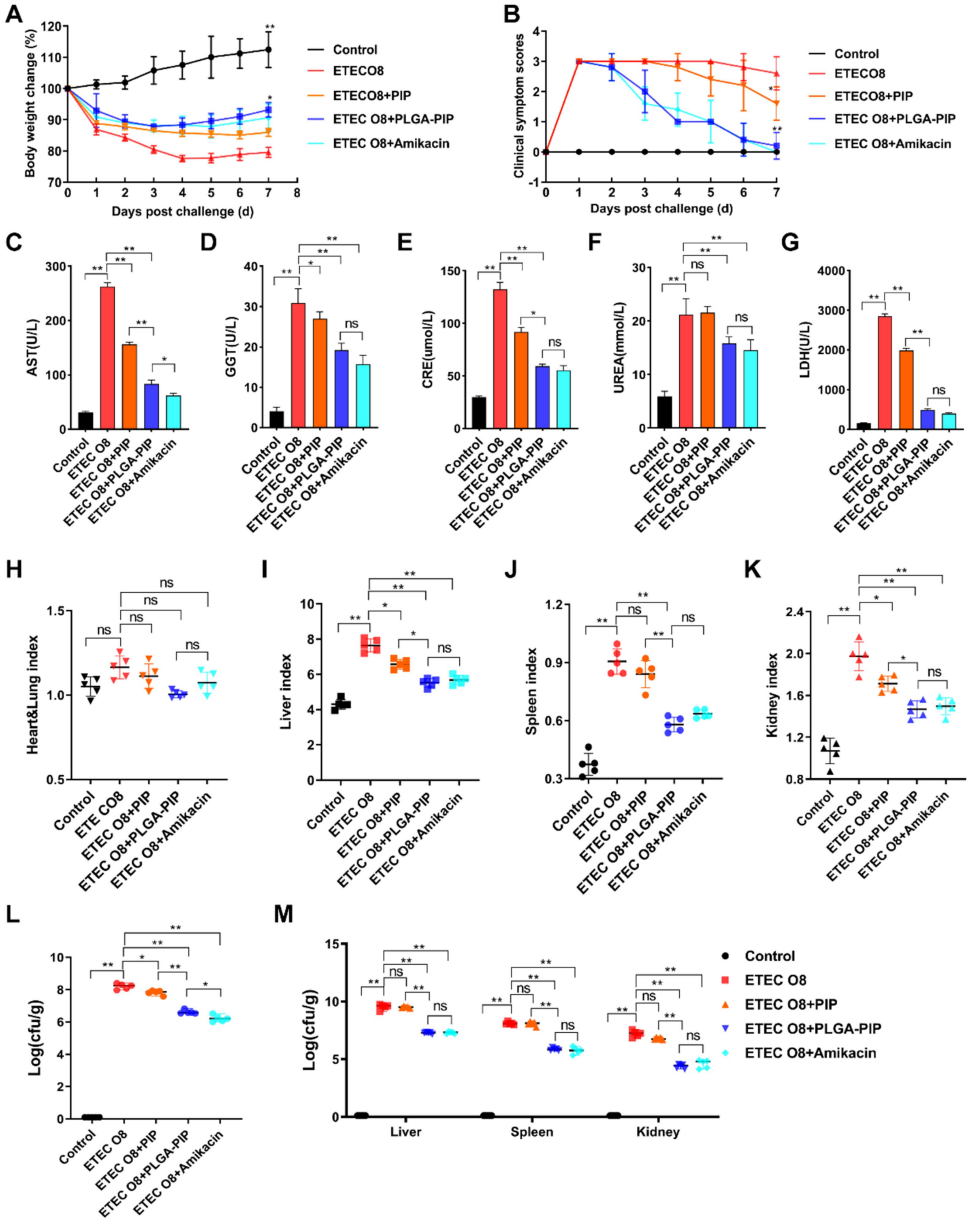
PLGA-PIP had a positive impact on the body weight, clinical symptoms, serum biochemical indicators, organ index, and bacterial load of mice infected with ETEC O8. **(A)** Clinical symptom score and **(B)** changes in body weight of mice infected with ETEC O8 after PLGA-PIP treatment. **(C)** AST, **(D)** GGT, **(E)** CRE, **(F)** urea, and **(G)** LDH levels in serum of mice infected with ETEC O8 after PLGA-PIP treatment. Effect of PLGA-PIP on the **(H)** heart and lung indexes, **(I)** liver index, **(J)** spleen index, and **(K)** kidney index of mice challenged with ETEC O8. **(L)** The number of bacteria in the feces of mice infected with ETEC O8 after PLGA-PIP treatment. **(M)** The number of bacteria in the liver, spleen and kidney of mice infected with ETEC O8 after PLGA-PIP treatment. **(A)** Error bars represent mean ± SD, *n* = 5. The differences between the groups were determined by one-way ANOVA followed by Tukey’s *post-hoc* analysis. ns represents no significant difference, ^*^p < 0.05 and ^**^p < 0.01. **(B–M)** Error bars represent median (IQR), *n* = 5. The differences between the groups were determined by Kruskal–Wallis *H* test followed by Dunn’s *post-hoc* test (nonparametric analysis for ordinal lesion scoring data) represents no significant difference, ^*^p < 0.05 and ^**^p < 0.01. PIP, PIL22-PBD-2.

The therapeutic effect of PLGA-PIP on ETEC O8 induced inflammation was evaluated in a Kunming mouse model, and the experimental design was shown in Figure 1. Clinical observation showed that the ETEC O8-infected mice exhibited fatigue, loss of appetite, clustering, messy fur, increased eye discharge, and severe diarrhea, which was consistent with the symptoms observed in the previous sepsis model. Compared with the ETEC O8 group, the above symptoms were significantly relieved in the PLGA-PIP group, and the therapeutic effect of PLGA-PIP was comparable to that of intra-abdominal antibiotics. The clinical symptom score of ETEC O8 infected mice is shown in Figure 6A. Additionally, PLGA-PIP reduced the weight loss of mice (Figure 6B). At 7 days post challenge (dpi), the degree of weight loss in the high-dose PLGA-PIP group of mice was significantly lower than that in the untreated group (Figure 6B, *p* < 0.01). There was no significant difference in the weight loss trend between the group treated with antibiotics.

After intraperitoneal infection with ETEC O8, the innate immune system recognizes pathogen-related molecular patterns, leading to inflammatory reactions that damage metabolic organs such as the liver and kidneys (9). In order to further investigate the inflammatory damage in mice and the alleviating effect of PLGA-PIP on inflammation, we measured the biochemical indicators AST (Figure 6C), GGT (Figure 6D), CRE (Figure 6E), URE (Figure 6F), and LDH (Figure 6G) in the serum, organ indices (Figures 6H–K), and bacterial load (Figures 6L–M) of mice at 7 dpi. The results showed that compared with the ETEC O8 group, the biochemical indexes were significantly decreased in both the PLGA-PIP group (*p* < 0.01) and the Amikacin group (*p* < 0.01). However, the treatment effect of PLGA-PIP is significantly better than that of PIP. Compared with the control group, mice injected intraperitoneally with ETEC O8 showed varying degrees of enlargement in the liver, spleen, and kidneys. The organ index of the livers, spleens, and kidneys in the ETEC O8 group was notably high (*p* < 0.01) and decreased after PLGA-PIP and Amikacin treatment (*p* < 0.01). The heart and lung weights were not significantly different after ETEC O8 infection (*p* > 0.05). Furthermore, PLGA-PIP treatment significantly reduced ETEC O8 content in mouse tissues (rectal contents, liver, spleen, and kidney) and was more effective than PIP treatment (Figures 6L–M). These results suggest that PLGA-PIP can effectively improve the survival rate of sepsis mice and alleviate bacterial infection.

### 3.6 PLGA-PIP improves intestinal morphology and reduces organ damage

**Figure 7:**
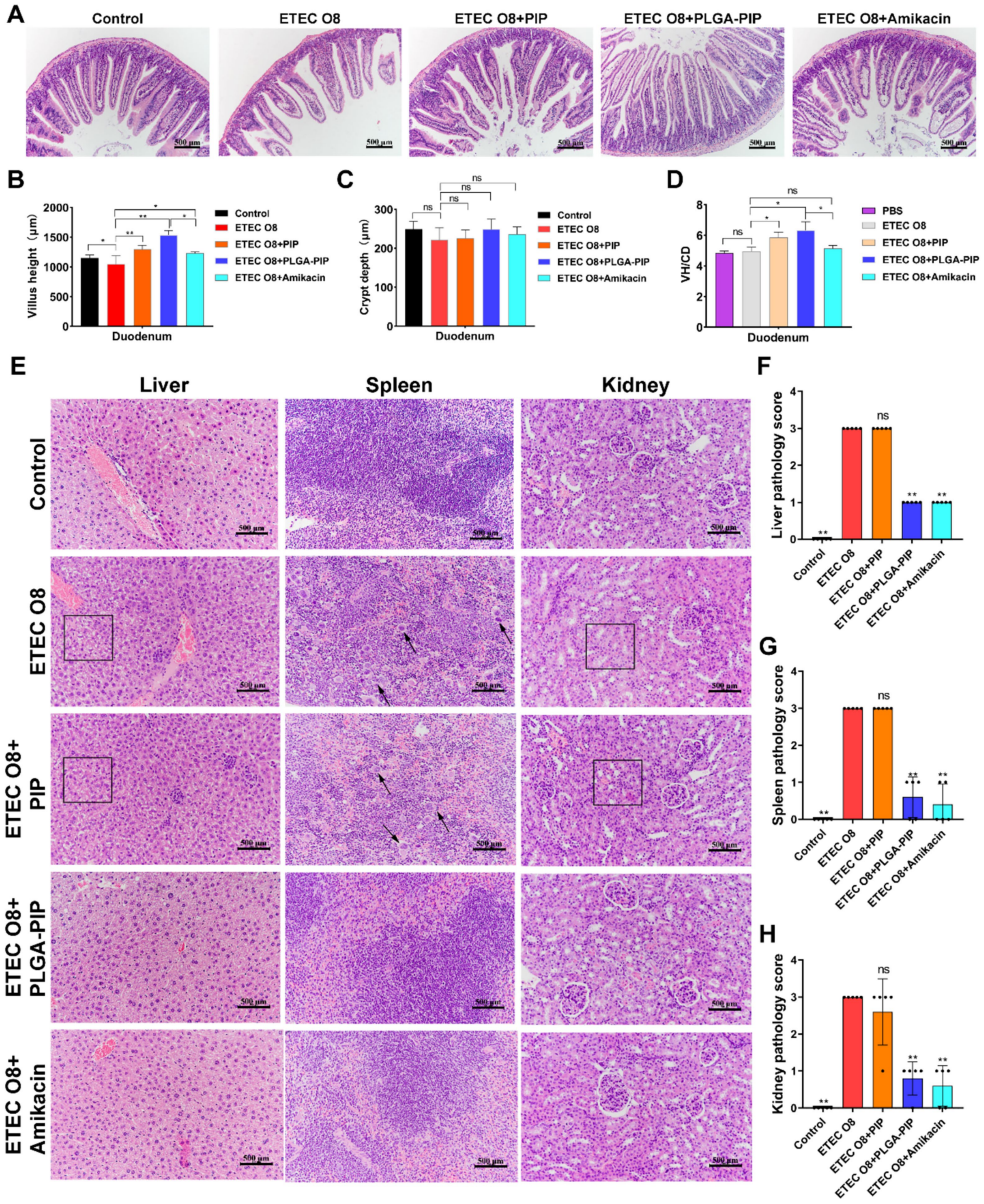
PLGA-PIP improves intestinal morphology and reduces organ damage. **(A)** The representative H&E staining photomicrographs of duodenal sections from each experimental group (bars, 500 μm). **(B)** Statistical analysis of duodenal villi height. **(C)** Statistical analysis of crypt depth. **(D)** The ratio of villi height to crypt depth (VH/CD). **(E)** Histopathological H&E staining of liver, spleen, and kidney tissues from each experimental group (bars, 500 μm). **(F)** Statistical analysis of liver pathological damage scores in each group. **(G)** Statistical analysis of splenic pathological damage scores in each group. **(H)** Statistical analysis of renal edema-associated pathological damage scores in each group. **(B–D)** Error bars represent mean ± SD, *n* = 5. The differences between the groups were determined by one-way ANOVA followed by Tukey’s *post-hoc* analysis. ns represents no significant difference, ^*^p < 0.05 and ^**^p < 0.01. **(F–H)** Error bars represent median (IQR), *n* = 5. The differences between the groups were determined by Kruskal–Wallis *H* test followed by Dunn’s *post-hoc* test (nonparametric analysis for ordinal lesion scoring data). ns represents no significant difference, ^*^p < 0.05 and ^**^p < 0.01. PIP, PIL22-PBD-2.

To assess the extent of tissue lesions, duodenum, liver, spleen, and kidney tissues were collected, fixed, and sliced for HE staining at 7 dpi. In intestinal sections, infection with ETEC O8 caused typical intestinal inflammation and barrier damage, intestinal villi shedding, reduced mucosal thickness, necrosis, large amounts of inflammatory cell infiltration into the duodenum, and disruption of intestinal villi (Figure 7A). The digestion and absorption of nutrients in mammals mainly occur in the small intestine (26). Villus height corresponds to the nutrient absorption area, and crypt depth corresponds to intestinal epithelium turnover (26). Therefore, villus height and crypt depth are regarded as indicators of intestinal health and development (26, 27). In comparison with the ETEC O8 group, the PLGA-PIP treatment increased the length of duodenal villi and the ratio of villi height to crypt depth in mice. These effects of PLGA-PIP were superior to those of the PIP group and the Amikacin group (Figures 7A–D). After being challenged by ETEC O8, the liver cells of mice showed obvious edema, blurred cell boundaries, liver cords, and irregular arrangement (Figure 7E,F). The splenic hyperemia and edema showed a large increase in macrophages (Figure 7E,G). The renal structure was slightly damaged, the renal parenchymal cells were showing edematous degeneration, and the renal tubular epithelial cells were also exhibiting edema (Figure 7E,H). Whereas PLGA-PIP and Amikacin better ameliorated the organs damage than PIP (Figure 7E). These results suggested that PLGA-PIP effectively improved the intestinal morphology and integrity and alleviated liver, spleen and kidney inflammation lesions in mice infected with ETEC O8.

### 3.7 PLGA-PIP inhibits inflammatory cytokine expression, maintains tight junction protein expression, and increases endogenous antimicrobial peptide expression in sepsis mice

**Figure 8:**
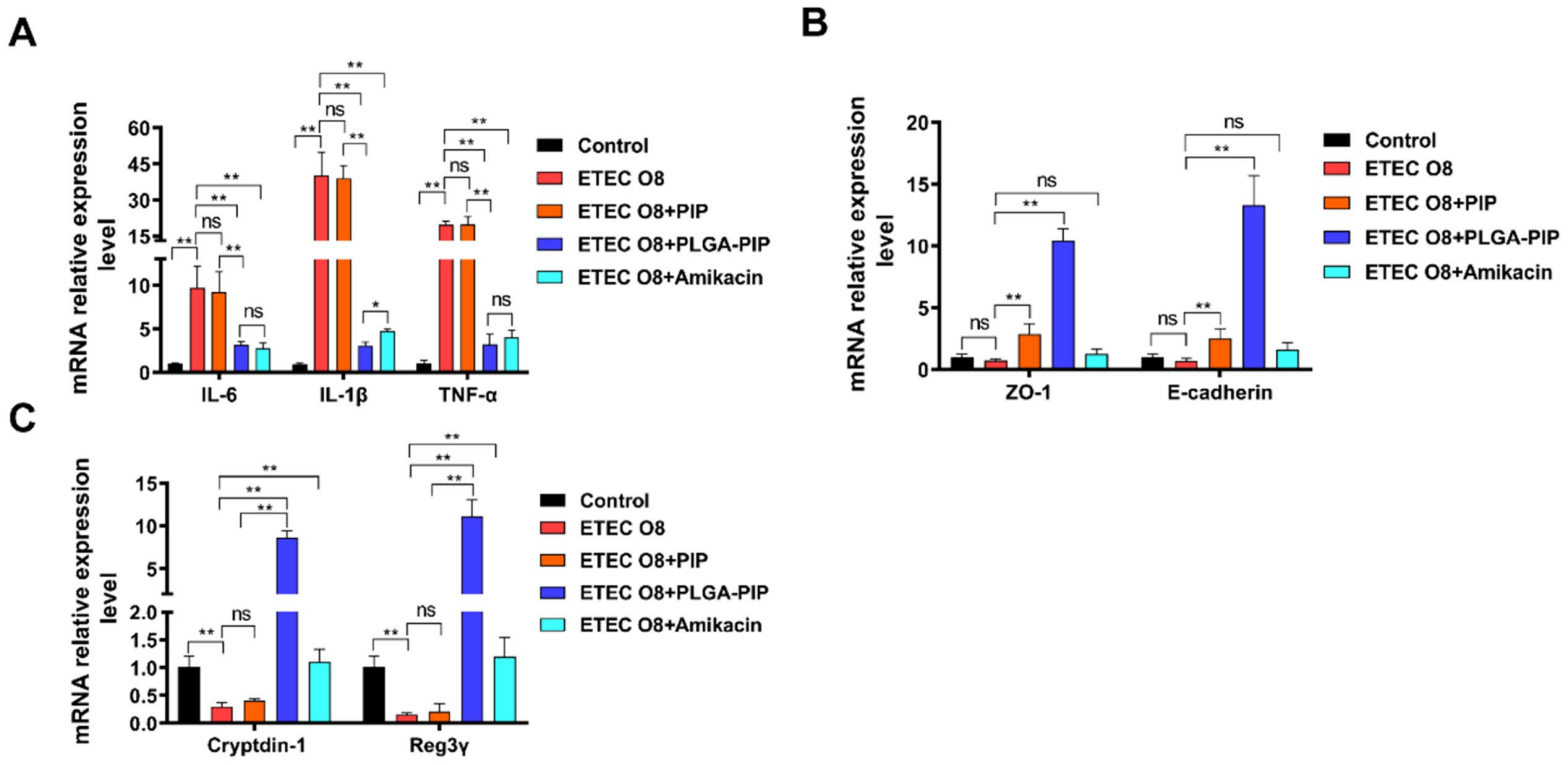
PLGA-PIP inhibits inflammatory cytokine expression, maintains tight junction protein expression, and increases endogenous antimicrobial peptide expression in sepsis mice. **(A)** Relative mRNA expression of IL-6, IL-1β, and TNF-α in duodenal tissues of each experimental group. **(B)** Relative mRNA expression of ZO-1 and E-cadherin in duodenal tissues of each experimental group. **(C)** Relative mRNA expression of Cyptdtin-1 and Reg3γ in duodenal tissues of each experimental group. Error bars represent mean ± SD. The differences between the groups were determined by one-way ANOVA followed by Tukey’s *post-hoc* analysis. ns represents no significant difference, ^*^p < 0.05 and ^**^p < 0.01. PIP, PIL22-PBD-2.

To further investigate the anti-inflammatory effect of PLGA-PIP, the mRNA expression of representative inflammatory factors including IL-1β, IL-6, and TNF-α in the duodenum was detected by real-time PCR after ETEC O8 challenge. In comparison with the control group, ETEC O8 infection significantly increased the expression of the inflammatory factors IL-6, IL-1β, and TNF-α in the duodenum (Figure 8A, *p* < 0.01). Treatment with PLGA-PIP and Amikacin significantly inhibited their mRNA expression in the duodenum (*p* < 0.01).

The normal intestinal barrier, formed by epithelial cells and the junctional complex, including tight junctions and adhesion junctions, plays an important role in the mucosal immunity, inflammation, and defense against the invasion of harmful substances and microbiota (14). Therefore, we next sought to clarify the effects of PLGA-PIP on the mRNA expression of gut barrier genes in duodenal tissues after ETEC O8 challenge. As shown in Figure 8B, Amikacin does not promote mRNA expression of cell junction proteins, including ZO-A and E-cadherin. Compared to the amikacin group, PIP and PLGA-PIP significantly increased the gene expression levels of ZO-1 and E-cadherin. In addition, PLGA-PIP can promote the expression of cell adhesion proteins more effectively than PIP. These results suggest that PLGA-PIP enhances intestinal barrier function by upregulating the mRNA expression of junction protein related genes.

In our previous studies, PIP not only directly plays a role in the bacterial infection process but also induces the production of endogenous defense molecules and promotes the integrity of IPEC-J2 cells (13). In this study, we found that PLGA-PIP significantly upregulated the gene expression levels of endogenous antimicrobial peptides Cryptdin-1 and Reg3γ in mouse intestines, helping mice resist colonization of ETEC O8 in the intestines (Figure 8C). Together, oral administration of PLGA-PIP can reduce the inflammatory damage caused by ETEC O8. This is achieved by increasing the mRNA expression of intestinal barrier protein and endogenous antimicrobial peptides, inhibiting the expression of inflammatory cytokines, and reducing tissue bacterial load and lesions.

## 4 Discussion

Facing detriment of livestock production caused by multidrug-resistant bacteria resulting from excessive antibiotics, the development of antibiotic substitutes with low cost, no obvious toxicity, and excellent therapeutic effect is a challenge and high priority (23, 28). As part of the body’s innate immunity, AMPs have the advantages of high efficiency, broad spectrum, and are not susceptible to bacterial resistance (29). Therefore, a great variety of AMPs have been developed as alternative antibiotics to treat bacterial infections (3, 9, 30). Our preliminary research demonstrated that compared to individual PBD2, the fusion expression of PBD2 with PIL22 not only exhibited broad-spectrum antibacterial activity, but also showed a significant healing effect on scratch wounds in IPEC-J2 cells (13). However, it remains unclear whether the recombinant protein PIP exerts its antibacterial and intestinal repair characteristics in animal models. In this study, the recombinant antimicrobial protein PIP was highly expressed in yeast and encapsulated into PLGA to form enzyme-resistant PLGA-PIP nanoparticles. Subsequently, we evaluated the safety and efficacy of PLGA-PIP and PIP against ETEC O8 infections in mice.

PLGA has excellent biocompatibility, biodegradability, biosafety, versatility, and both controlled and sustained release properties. It has been approved by the Food and Drug Administration and is widely considered to be the most successful polymer in the biomedical field (18, 31). In particular, the controlled release of the protein from the PLGA nano-systems overcomes the disadvantage of using peptide-based agents by ensuring that the protein is protected from hydrolytic and enzymatic degradation in the harsh gastric environment of the gastrointestinal (GI) tract through PLGA encapsulation (21, 31). The principle of PLGA binding to PIP has been confirmed. It is based on the fact that the negatively charged surface of PLGA can attract the positively charged PIP through electrostatic interaction. Subsequently, new nanoparticles are formed by shear forces (22, 23). In addition, the pores of PLGA allow for more efficient encapsulation of proteins (19). In this study, we successfully loaded PIP into PLGA nanoparticles and obtained PLGA-PIP nanoparticles. PLGA-PIP has uniform particle size, good biocompatibility, no obvious biotoxicity, and sustained release. Previous studies have shown that sustained release of nanoparticles can help AMPs exert a longer-lasting function both *in vitro* and *in vivo* (14, 23). The results of continuous release *in vitro* showed that PLGA-PIP nanoparticles also have sustained release properties, allowing them to be continuously released in gastric and intestinal fluid environments. Free proteins and peptides (PPs) were readily degraded in the GI tract by enzymes, greatly affecting the function of PPs (21, 32). In the Oxford cup bacteriostasis experiment, PLGA-PIP still exhibited antibacterial activity even after treatment with trypsin. This may be attributed to the protection of PIP within PLGA pores from trypsin-induced damage (14). More importantly, this sustained-release kinetic profile prevents the formation of transiently high free PIP concentrations in the intestinal lumen, which would otherwise enhance exposure to pancreatic enzymes and accelerate peptide degradation. In contrast, the gradual release of PIP maintains a pharmacologically effective local concentration while minimizing interactions with pancreatic enzymes in the bulk intestinal fluid.

Due to the recent expression of PIP, there has been a lack of research on its application *in vivo*. Our previous study found that PIP exhibits a significant inhibitory effect against both gram-positive and gram-negative bacteria, particularly targeting the multidrug-resistant ETEC O8 strain (13). However, it should be noted that we observed a decrease in the viability of IPEC-J2 cells following prolonged exposure to high doses of PIP (13). Consequently, it is imperative to conduct a prior evaluation of the safety profile of PIP *in vivo* before considering its application in mice. We set PIP at 75 mg/kg, 150 mg/kg, and 300 mg/kg based on the recommended therapeutic dose of amikacin (15 mg/kg) and the LE of PLGA-PIP for safety evaluation of PIP in mice. After a two-week administration of different doses of PLGA-PIP and PBS, weight monitoring results revealed no significant differences in body weight and daily gain between the 75 mg/kg and 150 mg/kg PLGA-PIP groups when compared with the PBS group. Notably, the body weight of the 300 mg/kg PLGA-PIP group exhibited an upward trend compared with the control group. The results demonstrated that the administration of 300 mg/kg PLGA-PIP did not have any detrimental impact on the growth of mice. In general, the liver and kidney are involved in drug metabolism, so indices of their function are important for assessing the toxicity of drugs in living organisms (14). The results of serum biochemical indexes showed no discernible disparities in serum CRE, AST, and UREA levels across all experimental groups. Additionally, there were also no lesions in the heart, liver, spleen, lung, and kidney tissues across all experimental groups. Based on previous and current research findings, we speculate that continuous feeding of a high dose of PLGA-PIP has no harmful effects on mice. This may be attributed to the following factors: on the one hand, PLGA is a copolymer of lactic acid and glycolic acid with excellent biocompatibility, biodegradability, low immunogenicity, and low toxicity (33); on the other hand, PIP is adsorbed on the surface and pores of PLGA and is continuously and slowly released, thus avoiding the simultaneous action of high-dose antibacterial proteins at the GI site. In summary, a dose of 300 mg/kg of PLGA-PIP was administered in the subsequent mouse infection experiments to achieve optimal therapeutic effects.

The selection of distinct administration routes was strictly based on the inherent pharmacokinetic properties of the tested agents and the practical needs of the study. Amikacin, as an aminoglycoside antibiotic, exhibits extremely low oral bioavailability owing to gastrointestinal degradation and first-pass metabolism. Intraperitoneal injection was therefore chosen to bypass these barriers, ensure sufficient systemic exposure, and guarantee reliable therapeutic efficacy, which aligns with the common practice of using parenteral routes for aminoglycosides to achieve effective drug concentrations (34). In contrast, PIL22-PBD2 and PLGA-PIL22-PBD2 are antimicrobial peptides (AMPs) developed for intestinal infection treatment. Oral administration via drinking water was selected to simulate clinical application scenarios for intestinal-targeted therapeutics, given that oral delivery represents the most convenient and patient-compliant route for such indications. Additionally, PLGA-based nanocarriers are widely utilized to enhance the oral bioavailability of AMPs by protecting them from gastrointestinal enzymatic degradation. To minimize potential confounding effects associated with the different administration routes, we strictly standardized all critical experimental variables across all groups. These included consistent intervention duration, unified sampling time points, and identical core efficacy evaluation endpoints, specifically intestinal ETEC O8 load, fecal bacterial colony enumeration, and relief of clinical symptoms. These endpoints directly reflect the therapeutic effect on intestinal ETEC infection, thus ensuring the comparability of efficacy data between groups.

Because most ETEC strains are host-specific, it is difficult to establish a mouse pathogenic model by orally administering *E. coli* (35), and a similar situation exists for enterohemorrhagic *E. coli* (36, 37). In the present study, we established a sepsis model in mice exhibiting diarrhea symptoms through intraperitoneal injection. Mice challenged with ETEC O8 showed swollen conjunctiva with secretions, lethargy, loss of appetite, messy back hair, and diarrhea, consistent with a previous study (25). Many studies have shown that AMPs have a significant therapeutic effect on *E. coli*-induced sepsis in mice (5, 9, 25). For example, an AMP, AMPR-11, derived from the non-selective channel romo1 of mitochondria, showed significant therapeutic effects against bacteria (including multidrug-resistant strains) in a mouse sepsis model after intravenous administration. The peptide also increased the survival rate of septic mice caused by multidrug-resistant bacteria (5). Our research shows that the oral administration of PLGA-PIP can significantly improve the clinical status of septic mice, restore organ indices, reduce organ bacterial load, and alleviate organ damage. However, the therapeutic effect of orally feeding PIP to septic mice was not significant.

One of the pathogeneses of systemic inflammation caused by bacteria invading the abdominal cavity is the uncontrolled inflammatory response and immune dysfunction triggered by endotoxin released by gram-negative bacteria (5, 9). Systemic inflammation leads to an imbalance in intestinal homeostasis. ETEC O8 produces toxins, which cause damage to the intestinal mucosal barrier and result in diarrhea (25). In this study, the duodenal tissue of mice in the ETEC O8 group showed increased permeability and inflammatory damage. Histological sections of the tissue revealed damaged duodenal mucosa, decreased gland numbers, and irregular or missing intestinal villi. Additionally, the villus height and the VH/CD ratio decreased statistically compared to the control group. This impaired intestinal morphology reduced the absorptive area of the small intestine and decreased the ability to absorb nutrients, which might have contributed to the significantly lower body weight in the ETEC O8 group (38). In comparison, oral administration of PLGA-PIP significantly reduces intestinal damage caused by ETEC O8, increasing villi height, and restores the VH/CD ratio to that in the PBS group. Long-term and excessive production of proinflammatory cytokines may lead to induced atrophy of intestinal mucosa and disruption of intestinal functions (39, 40). In this study, oral administration of PLGA-PIP significantly reduced the expression levels of inflammatory cytokines IL-1β, IL-6, and TNF-α, and increased the expression levels of endogenous antimicrobial peptides Reg3γ and Cryptdin-1 in the intestines of ETEC O8-infected mice. Additionally, PLGA-PIP alleviates intestinal barrier dysfunction by upregulating the mRNA expression of the tight junction protein ZO-1 and the adhesion molecule E-cadherin, thereby reducing intestinal permeability (41). Notably, compared to PIP, PLGA-PIP demonstrated greater efficacy in reducing intestinal permeability, decreasing the expression of intestinal inflammatory factors, and increasing the expression levels of endogenous antimicrobial peptides. This may be attributed to the potential damaging effects of PIP in the complex gastrointestinal environment, which have impacted the effectiveness of the treatment (21). Additionally, PLGA nanoparticles are known to exhibit intestinal mucosa-adherent properties, which promotes the enrichment of PLGA-PIP on the intestinal epithelial surface, the primary target site of ETEC O8 infection. This mucosal localization not only enhances the interaction between released PIP and intestinal epithelial cells (facilitating antibacterial activity and barrier repair) but also reduces the diffusion of free PIP into the intestinal lumen. These results are consistent with another study on the treatment of ulcerative colitis using mesoporous carbon nanoparticles (MCNs) loaded with *Musca domestica* cecropin (MDC). Specifically, the therapeutic effect of orally administering MCN-MDC is superior to using MDC alone (14). These results highlight the advantages of PLGA delivery systems, which can help protect naked PIP from damage in the complex environment of the GI tract and slowly release PIP to establish long-lasting therapy in the intestines.

## 5 Conclusion

Collectively, the PLGA nano-delivery system ensures the *in vivo* stability of PIP through two key synergistic effects: pre-release protection via the polymer barrier during gastrointestinal transit, and post-release stability enhancement via sustained release and mucosal localization that reduces exposure to pancreatic enzymes. This mechanism is consistent with our *in vitro* pancreatic enzyme resistance data and *in vivo* therapeutic outcomes, validating the rational design of the PLGA-PIP nanoformulation for intestinal pathogenic *E. coli* infection. The ability to overcome peptide degradation by pancreatic enzymes highlights the advantage of PLGA as a delivery carrier for peptide-based antimicrobials, providing a viable strategy to address the inherent limitations of AMPs in oral application.

## Data Availability

The raw data supporting the conclusions of this article will be made available by the authors, without undue reservation.

## References

[1] Li Q Dai JJ Chen SY Sun RY Wang D Bai SC . Prevalence and molecular characteristics of intestinal pathogenic *Escherichia coli* isolated from diarrheal pigs in southern China. Vet Microbiol. (2024) 296:110171. doi: 10.1016/j.vetmic.2024.110171, 38981202

[2] Tang Q Lan T Zhou C Gao J Wu L Wei H . Nutrition strategies to control post-weaning diarrhea of piglets: from the perspective of feeds. Anim Nutr. (2024) 17:297–311. doi: 10.1016/j.aninu.2024.03.006, 38800731 PMC11127239

[3] Xie K Xie H Su G Chen D Yu B Mao X . β-Defensin 129 attenuates bacterial endotoxin-induced inflammation and intestinal epithelial cell apoptosis. Front Immunol. (2019) 10:2333. doi: 10.3389/fimmu.2019.02333, 31636641 PMC6787771

[4] Ma X Zhang Y Xu T Qian M Yang Z Zhan X . Early-life intervention using exogenous fecal microbiota alleviates gut injury and reduce inflammation caused by weaning stress in piglets. Front Microbiol. (2021) 12:671683. doi: 10.3389/fmicb.2021.671683, 34177852 PMC8222923

[5] Lee HR You DG Kim HK Sohn JW Kim MJ Park JK . Romo1-derived antimicrobial peptide is a new antimicrobial agent against multidrug-resistant bacteria in a murine model of sepsis. mBio. (2020) 11:e03258-19. doi: 10.1128/mBio.03258-19, 32291307 PMC7157825

[6] Zhang H Du Y Guo Y Wang Z Li H Lv Z . TLR4-NLRP3-GSDMD-mediated pyroptosis plays an important role in aggravated liver injury of CD38^−/−^ sepsis mice. J Immunol Res. (2021) 2021:6687555. doi: 10.1155/2021/6687555, 33860064 PMC8026301

[7] Huang C Yang X Huang J Liu X Yang X Jin H . Porcine beta-defensin 2 provides protection against bacterial infection by a direct bactericidal activity and alleviates inflammation via interference with the TLR4/NF-κB pathway. Front Immunol. (2019) 10:1673. doi: 10.3389/fimmu.2019.01673, 31379864 PMC6657668

[8] Liu Y Gao Z Chen T Gao Y Chen H Ye H . Versatile fabrication of biocompatible antimicrobial materials enabled by cationic peptide bundles. ACS Appl Mater Interfaces. (2024) 16:43918–32. doi: 10.1021/acsami.4c06381, 39126384

[9] Tan P Tang Q Xu S Zhang Y Fu H Ma X. Designing self-assembling chimeric peptide nanoparticles with high stability for combating piglet bacterial infections. Adv Sci. (2022) 9:e2105955. doi: 10.1002/advs.202105955, 35285170 PMC9109057

[10] Califano D Cho JJ Uddin MN Lorentsen KJ Yang Q Bhandoola A . Transcription factor Bcl11b controls identity and function of mature type 2 innate lymphoid cells. Immunity. (2015) 43:354–68. doi: 10.1016/j.immuni.2015.07.005, 26231117 PMC4657441

[11] Li Y Wang J Li Y Wu H Zhao S Yu Q. Protecting intestinal epithelial cells against deoxynivalenol and *E. coli* damage by recombinant porcine IL-22. Vet Microbiol. (2019) 231:154–9. doi: 10.1016/j.vetmic.2019.02.027, 30955803 PMC7172643

[12] Hendrikx T Duan Y Wang Y Oh JH Alexander LM Huang W . Bacteria engineered to produce IL-22 in intestine induce expression of REG3G to reduce ethanol-induced liver disease in mice. Gut. (2019) 68:1504–15. doi: 10.1136/gutjnl-2018-317232, 30448775 PMC6387784

[13] Li X Qiu P Yue M Zhang Y Lei C Wang J . Expression of recombination antimicrobial protein PIL22-PBD-2 in Pichia pastoris and verification of its biological function *in vitro*. Vet Res. (2025) 56:52. doi: 10.1186/s13567-024-01428-1, 40055823 PMC11889930

[14] Zhang L Gui S Xu Y Zeng J Wang J Chen Q . Colon tissue-accumulating mesoporous carbon nanoparticles loaded with *Musca domestica* cecropin for ulcerative colitis therapy. Theranostics. (2021) 11:3417–38. doi: 10.7150/thno.53105, 33537095 PMC7847694

[15] Jana D Han Z Huang X Wadhwa A Raveendran A Ebeid K . Enhanced prostate-specific membrane antigen targeting by precision control of DNA scaffolded nanoparticle ligand presentation. ACS Nano. (2024) 18:16674–83. doi: 10.1021/acsnano.4c01640, 38907991 PMC11223598

[16] Zuo S Wang Z Jiang X Zhao Y Wen P Wang J . Regulating tumor innervation by nanodrugs potentiates cancer immunochemotherapy and relieve chemotherapy-induced neuropathic pain. Biomaterials. (2024) 309:122603. doi: 10.1016/j.biomaterials.2024.122603, 38713972

[17] Yan Y Huang X Yuan L Ngai T Ma G Xia Y. Dictating the spatial-temporal delivery of molecular adjuvant and antigen for the enhanced vaccination. Biomaterials. (2024) 311:122697. doi: 10.1016/j.biomaterials.2024.122697, 38968687

[18] Pinto M Silva V Barreiro S Silva R Remião F Borges F . Brain drug delivery and neurodegenerative diseases: polymeric PLGA-based nanoparticles as a forefront platform. Ageing Res Rev. (2022) 79:101658. doi: 10.1016/j.arr.2022.101658, 35660114

[19] Wang M Wang S Zhang C Ma M Yan B Hu X . Microstructure formation and characterization of long-acting injectable microspheres: the gateway to fully controlled drug release pattern. Int J Nanomedicine. (2024) 19:1571–95. doi: 10.2147/ijn.S445269, 38406600 PMC10888034

[20] Danhier F Ansorena E Silva JM Coco R Le Breton A Préat V. PLGA-based nanoparticles: an overview of biomedical applications. J Control Release. (2012) 161:505–22. doi: 10.1016/j.jconrel.2012.01.043, 22353619

[21] Zhu Q Chen Z Paul PK Lu Y Wu W Qi J. Oral delivery of proteins and peptides: challenges, status quo and future perspectives. Acta Pharm Sin B. (2021) 11:2416–48. doi: 10.1016/j.apsb.2021.04.001, 34522593 PMC8424290

[22] Lee SE Lee CM Won JE Jang G-Y Lee JH Park SH . Enhancement of anticancer immunity by immunomodulation of apoptotic tumor cells using annexin A5 protein-labeled nanocarrier system. Biomaterials. (2022) 288:121677. doi: 10.1016/j.biomaterials.2022.121677, 35927088

[23] Huo X Wang Z Xiao X Yang C Su J. Oral Administration of Nanopeptide CMCS-20H conspicuously boosts immunity and precautionary effect against bacterial infection in fish. Front Immunol. (2021) 12:811616. doi: 10.3389/fimmu.2021.811616, 35087530 PMC8786714

[24] Freitas R Ferreira E Miranda A Ferreira D Relvas-Santos M Castro F . Targeted and self-adjuvated nanoglycovaccine candidate for cancer immunotherapy. ACS Nano. (2024) 18:10088–103. doi: 10.1021/acsnano.3c12487, 38535625

[25] Zhao X Wang L Zhu C Xia X Zhang S Wang Y . The antimicrobial peptide mastoparan X protects against enterohemorrhagic *Escherichia coli* O157:H7 infection, inhibits inflammation, and enhances the intestinal epithelial barrier. Front Microbiol. (2021) 12:644887. doi: 10.3389/fmicb.2021.644887, 34177825 PMC8222680

[26] Salahuddin M Hiramatsu K Tamura K Kita K. Dietary carbohydrate effects on histological features of ileal mucosa in White Leghorn chicken. J Vet Med Sci. (2021) 83:952–6. doi: 10.1292/jvms.21-0157, 33883363 PMC8267207

[27] Feng L Ye W Zhang K Qu D Liu W Wu M . *In vitro* digestion characteristics of hydrolyzed infant formula and its effects on the growth and development in mice. Front Nutr. (2022) 9:912207. doi: 10.3389/fnut.2022.912207, 35811942 PMC9263559

[28] Sabino YNV Santana MF Oyama LB Santos FG Moreira AJS Huws SA . Characterization of antibiotic resistance genes in the species of the rumen microbiota. Nat Commun. (2019) 10:5252. doi: 10.1038/s41467-019-13118-0, 31748524 PMC6868206

[29] Chen J Wang W Hu X Yue Y Lu X Wang C . Medium-sized peptides from microbial sources with potential for antibacterial drug development. Nat Prod Rep. (2024) 41:1235–63. doi: 10.1039/d4np00002a, 38651516

[30] Ouyang M Wu F Hu C. Efficacy of short novel antimicrobial peptides in a mouse model of *Staphylococcus pseudintermedius* skin infection. Antibiotics. (2024) 13:508. doi: 10.3390/antibiotics13060508, 38927175 PMC11200854

[31] Akmayan İ Oztav S Coksu I Abamor ES Acar S Ozbek T. Construction of recombinant Omp25 or EipB protein loaded PLGA nanovaccines for brucellosis protection. Nanotechnology. (2024) 35:395707. doi: 10.1088/1361-6528/ad5b66, 38917779

[32] Moscoso-Mujica G Zavaleta AI Mujica Á Arnao I Moscoso-Neira C Santos M . Antimicrobial peptides purified from hydrolysates of kanihua (*Chenopodium pallidicaule* Aellen) seed protein fractions. Food Chem. (2021) 360:129951. doi: 10.1016/j.foodchem.2021.129951, 33989882

[33] Shen AM Minko T. Pharmacokinetics of inhaled nanotherapeutics for pulmonary delivery. J Control Release. (2020) 326:222–44. doi: 10.1016/j.jconrel.2020.07.011, 32681948 PMC7501141

[34] Yu J Tang H Chen Y Wang Z Huang W Zhou T . Salmonella utilizes L-arabinose to silence virulence gene expression for accelerated pathogen growth within the host. Gut Microbes. (2025) 17:2467187. doi: 10.1080/19490976.2025.2467187, 39954030 PMC11834461

[35] Carroll CJ Hocking DM Azzopardi KI Praszkier J Bennett-Wood V Almeida K . Re-evaluation of a neonatal mouse model of infection with enterotoxigenic *Escherichia coli*. Front Microbiol. (2021) 12:651488. doi: 10.3389/fmicb.2021.651488, 33815340 PMC8013722

[36] Bowser S Melton-Celsa A Chapartegui-González I Torres AG. Further evaluation of enterohemorrhagic *Escherichia coli* gold nanoparticle vaccines utilizing *Citrobacter rodentium* as the model organism. Vaccine. (2024) 12:508. doi: 10.3390/vaccines12050508, 38793759 PMC11125983

[37] Ritchie JM. Animal models of enterohemorrhagic *Escherichia coli* infection. Microbiol Spectr. (2014) 2:Ehec-0022-2013. doi: 10.1128/microbiolspec.EHEC-0022-2013 26104195

[38] Li W Zeng Z Zhou D Wang G Wang Z Li Y . Effect of oral administration of microcin Y on growth performance, intestinal barrier function and gut microbiota of chicks challenged with *Salmonella* Pullorum. Vet Res. (2024) 55:66. doi: 10.1186/s13567-024-01321-x, 38778424 PMC11112776

[39] Sanchez-Munoz F Dominguez-Lopez A Yamamoto-Furusho JK. Role of cytokines in inflammatory bowel disease. World J Gastroenterol. (2008) 14:4280–8. doi: 10.3748/wjg.14.4280, 18666314 PMC2731177

[40] Lee SH Lillehoj HS Jang SI Lillehoj EP Min W Bravo DM. Dietary supplementation of young broiler chickens with Capsicum and turmeric oleoresins increases resistance to necrotic enteritis. Br J Nutr. (2013) 110:840–7. doi: 10.1017/s0007114512006083, 23566550

[41] Castro-Ochoa KF Vargas-Robles H Chánez-Paredes S Felipe-López A Cabrera-Silva RI Shibayama M . Homoectoine protects against colitis by preventing a claudin switch in epithelial tight junctions. Dig Dis Sci. (2019) 64:409–20. doi: 10.1007/s10620-018-5309-8, 30269272

[42] Cheng F Qiao Z Liang G Li J Qiao Y Yun S . Polysaccharide from *Sparassis latifolia* alleviates intestinal barrier dysfunction in mice exposed to lead. Int J Biol Macromol. (2023) 253:127615. doi: 10.1016/j.ijbiomac.2023.127615, 37879574

[43] Li CL Tan LH Wang YF Luo CD Chen HB Lu Q . Comparison of anti-inflammatory effects of berberine, and its natural oxidative and reduced derivatives from Rhizoma Coptidis *in vitro* and *in vivo*. Phytomedicine. (2019) 52:272–83. doi: 10.1016/j.phymed.2018.09.228, 30599908

